# Regulation and Trust: 3-Month Follow-up Study on COVID-19 Mortality in 25 European Countries

**DOI:** 10.2196/19218

**Published:** 2020-04-24

**Authors:** Atte Oksanen, Markus Kaakinen, Rita Latikka, Iina Savolainen, Nina Savela, Aki Koivula

**Affiliations:** 1 Tampere University Tampere Finland; 2 University of Helsinki Helsinki Finland; 3 University of Turku Turku Finland

**Keywords:** mortality, infectious diseases, sociability, trust, prevention, Europe

## Abstract

**Background:**

The outbreak of the coronavirus disease (COVID-19) has dramatically changed societies in 2020. Since the end of February, Europe has been hit particularly hard by COVID-19, but there are major country differences in both the spread of the virus and measures taken to stop the virus. Social psychological factors such as institutional trust could be important in understanding the development of the epidemic.

**Objective:**

The aim of this study was to examine country variations of COVID-19 mortality in Europe by analyzing social risk factors explaining the spread of the disease, restrictions and control measures, and institutional trust.

**Methods:**

The present study was based on a background analysis of European Social Survey data on 25 European countries (N=47,802). Multilevel mixed effects linear regression models focused on 84 days of the COVID-19 epidemic (January 22 to April 14, 2020) and modelled the daily COVID-19 mortality. Analysis focused on the impact of social relations, restrictions, and institutional trust within each country.

**Results:**

The spread of the COVID-19 epidemic has been fast everywhere, but the findings revealed significant differences between countries in COVID-19 mortality. Perceived sociability predicted higher COVID-19 mortality. Major differences between the 25 countries were found in reaction times to the crisis. Late reaction to the crisis predicted later mortality figures. Institutional trust was associated with lower COVID-19 mortality.

**Conclusions:**

The analyses demonstrated the importance of societal and social psychological factors in the spread of the COVID-19 epidemic. By considering multiple perspectives, this study showed that country differences in Europe are major, and this will have an impact on how countries will cope with the ongoing crisis in the following months. The results indicated the importance of timely restrictions and cooperation with people.

## Introduction

The worldwide outbreak of a new type of coronavirus (severe acute respiratory syndrome [SARS] coronavirus 2) causing coronavirus disease (COVID-19) has rapidly changed societies in the first 3 months of 2020. COVID-19 was first reported in December 2019 in Wuhan, the capital of Hubei Province, China [1]. As a response to a broader disease threat, China placed restrictions on travel in and out of Wuhan on January 23, 2020, but the virus was detected in Europe already in January in countries such as France (January 24, 2020) and Finland (January 26, 2020) [2]. Currently, it is not known how long there were active COVID-19 cases circling in Europe before different countries started to react to the epidemic. The first death caused by COVID-19 outside Asia occurred in France on February 15, 2020. In Italy, the number of infections started to rise rapidly in the last week of February [3]. During March 2020, almost all European countries placed at least some restrictions in an effort to prevent a further uncontrolled spread of the virus.

Much of the focus of COVID-19 discussion and research has centralized on epidemiological factors. The reproductive number (R_0_) of COVID-19 has been considered higher than that of SARS. In a recent review study, the average R_0_ of COVID-19 was found to be 3.28 with a median of 2.79 [4]. Viral shedding of the novel coronavirus is also long (median 20 days in survivors), and nonsurvivors have died, on average, after 18-19 days of illness onset [5,6]. Case fatality and infection fatality ratios have been recently reported for China, being 3.67% and 0.66%, respectively [6]. In Europe, similar estimations have not been made yet, but COVID-19 mortality has been particularly high in some regions such as Lombardy, Italy. Data shows major country variations in the spread and mortality rates of COVID-19 within Europe, but reasons behind the spread of the disease and subsequent mortality remain partly unexplained. Different countries have also responded to the epidemic at different rates, which gives a starting point for our investigations on societal and psychological factors related to the spread of COVID-19. A social scientific perspective could help us understand COVID-19 mortality.

Social factors are important in epidemics, which should always be understood in their ecological context [7]. This means, for example, that social activity has an impact on the spread of viruses. European countries vary greatly in terms of population density, and there are also differences in the number of social contacts people have and interact with on a daily basis. In addition, there are major cultural differences in the physical distance people keep when interacting with their close friends and other people [8]. For instance, Southern European countries have been traditionally considered as contact cultures in comparison to noncontact cultures, such as North Europe and Asia [8-10]. During an epidemic, both the physical and social closeness of people are factors that explain the spread of the disease.

Another important social factor explaining the spread of viruses is trust. Trust in institutions and other people is considered an important factor in the well-being and overall functioning of societies [11,12]. Institutional trust can be a crucial part of epidemic management and prevention because trust in public systems and authorities such as health care systems influences how people use services and follow instructions [13]. Trust in institutions becomes important after disruptive events such as terrorist attacks, natural disasters, or epidemics [14,15]. Research evidence from previous epidemics showed that those who had lower trust in the government were less likely to take precautions against the Ebola virus disease in Liberia and Congo during the 2014-2016 outbreak [16,17]. Similar findings were also noted during the 2002-2004 SARS outbreak in Hong Kong [18]. Great trust in authorities has also been associated with carrying out avoidant behaviors during the swine flu epidemic in the United Kingdom [19].

Dozens of studies have previously demonstrated significant country differences in institutional trust, making it an essential societal element to consider [20,21]. Trust in state institutions is typically highest in Nordic countries (Finland, Denmark, Iceland, Norway, Sweden), which also rank high in different welfare statistics worldwide [22]. Elsewhere in Europe, institutional trust is found to be low, particularly in Eastern European countries but also in Southern European countries such as Italy [23,24]. Determinants of institutional trust vary across different sides of Europe, but the perceived lack of responsiveness of political and governmental entities often results in low received trust from the public. In East Central Europe, older individuals and women have been found to show more trust toward institutions, while trust in political institutions is lower among more educated people [25]. In Southern European countries such as Italy and Spain, socialization experiences are largely associated with low institutional trust, and attitudes toward political institutions are deeply rooted in cultural legacy [26]. In other words, institutional trust is lowest in those countries characterized as contact cultures. The combination of social closeness and lack of trust in authorities might turn out to be lethal within Europe, at least for older adults.

The aim of our study was to examine country variations of COVID-19 mortality in Europe by analyzing social risk factors that may explain the spread of the disease, restrictions and control measures, and institutional trust. We expected to find societal differences especially in the capability of coping with this crisis situation.

## Methods

### Data Sources

This study was based on an analysis of European Social Survey (ESS) data on 25 European countries (N=47,802). Data were from 2016 (ESS8) except for Bulgaria, Cyprus, and Slovakia, whose data were from 2012 (ESS6), and Denmark with data from 2014 (ESS4). ESS data sets are openly available for research purposes at the ESS web site [27]. Additional country information was received from Eurostat and the World Bank. COVID-19 mortality and incidence figures were drawn from the database built by the Coronavirus Resource Centre at Johns Hopkins University [28]. The data were updated April 15, 2020, for this article. Country restrictions were drawn from the official websites of states and ministries, and other related webpages created for the purpose of providing COVID-19 updates.

### Ethics and Open Data

ESS data are publicly available and downloadable at the ESS website. The collection of their self-reported data is based on informed consent and subscribes to the Declaration of Professional Ethics of the International Statistical Institute. All ESS surveys have gone through ethical review by the ESS European Research Infrastructure Consortium Research Ethics Board [29]. Our analyses focused on creating country-level information, and no observations at the individual level were used. Other used data were also publicly available. All data and code are available via Open Science Framework [30].

### Measures

COVID-19 mortality and incidence time series data were collected for 25 European countries and covered 84 days of the COVID-19 epidemic (January 22 to April 14, 2020). Incidence rates were also collected, but they are treated only as controls, because countries differ a lot in their testing rates. Hence, mortality figures provide more accurate information on the spread of the epidemic from February to April 2020.

Information on country restrictions included national bans or restrictions. These included bans on public events, curfews, country border closures, restrictions on restaurant operations, and elementary school contact teaching. Public events, curfews, or unauthorized outings were reviewed and applied from the date when the first nationwide restriction became effective. Country border closures were determined starting from the date when all the borders of the country were closed. Restrictions on restaurant operations and elementary school contact teaching were calculated from the date when at least some national restrictions became effective. Restrictions varied in exact content and accuracy across countries.

General country information includes the size of the population, population density (persons per square kilometer), old-age dependency ratio (ie, ratio of people aged 65 years or older), gender ratio, life expectancy at birth, health care expenditure (euros per inhabitant), and number of tourist arrivals per year. Self-reported country information included perceived sociability, household size, the proportion of older people living with children, and perceived institutional trust.

The *perceived sociability* was measured with a question: “How often do you take part in social activities compared to others of same age.” The given responses were 1, “Much less than most,” 2 “Less than most,” 3 “About the same,” 4 “More than most,” and 5 “Much more than most.” *Household size* was based on respondents’ information on how many people live regularly in their household. *The proportion of older adults living with children* was calculated by grouping respondents aged 65 years or older according to whether they currently live in the same household with children. *Institutional trust* was measured by respondents’ trust in five institutions, namely, parliament, politicians, political parties, the police, and the legal system. Respondents were asked how much they personally trust these institutions on a scale from 0 to 10, in which 0 meant no trust at all, and 10 meant complete trust. Reliability of the measure was good with Cronbach alpha ranging from 0.82 to 0.92. In the analyses, institutional trust was categorized as very low (19 or less), low (20-22), high (23-29), and very high (30 or more) for an illustrative map, and as low (less than 23) and high (23 or more) based on the median for a random effects regression model.

### Statistical Techniques

All statistical analyses were conducted with Stata 16 software (StataCorp). Daily COVID-19 mortality during the COVID-19 epidemic in Europe was analyzed with multilevel mixed effects linear regression models. In the multilevel models, the dependent variable was the square root transformed daily mortality count. The count was based on daily follow-ups on COVID-19 mortality cases for each country, starting from the first confirmed infection and ending April 14, 2020. This resulted in follow-up periods that varied between countries (from 82 days in France to 37 days in Cyprus).

To assess the relationship between the daily mortality count and our main theoretical variables, we conducted three separate models: model 1 included perceived sociability, model 2 included timing of national restrictions, and model 3 included institutional trust as an independent variable. All models controlled for the following between-country factors: average household size, population, population density, old-age dependency ratio, life expectancy at birth, health care expenditure per inhabitant, high tourist arrival (dummy variable based on median), and the length of the follow-up period for each country. In addition, our models included time as a within-country predictor of mortality. The end point of our follow-up period (April 14, 2020) was coded as the zero point for our time variable. Preceding days had negative values in descending order until the first day of the country’s follow-up period. Thus, time was used to estimate the within-country change in mortality during the epidemic, and the between-country variables estimated the country differences in mortality. Except time and high tourist-arrival dummy variables, all independent variables were mean centered before adding them into the regression models.

All models were conducted with maximum likelihood estimation. We estimated Huber-White standard errors that were robust to within-country clustering and modelled our residuals to account for the autocorrelated error structure of our longitudinal data. The models included random intercept and random slope for time with unstructured covariances. We reported regression coefficients and corresponding 95% confidence intervals and *P* values for the fixed part of our models, and standard deviation with 95% confidence interval for the random effects.

The progression of COVID-19 mortality before and after the first COVID-19 death were analyzed with random effects models to account for clustering at the country level. We modelled the amount of daily deaths in low and high institutional trust (cutoff point median value 23) after the first COVID-19 death (time=0), which was used as a reference category. We then analyzed countries reacting late (restrictions placed after the first COVID-19 death) and early (restrictions placed before the first COVID-19 death). In both analyses each time point (day) was allowed to have a separate coefficient for the COVID-19 mortality value (presented as deaths/million persons). Models are presented as figures, and they are adjusted for population density, gender, old-age ratio, the proportion of those 65 years or older living with children, life expectancy, and tourist arrivals. Models included country restrictions as daily varying dummies (0=no control, 1=control).

## Results

**Figure 1:**
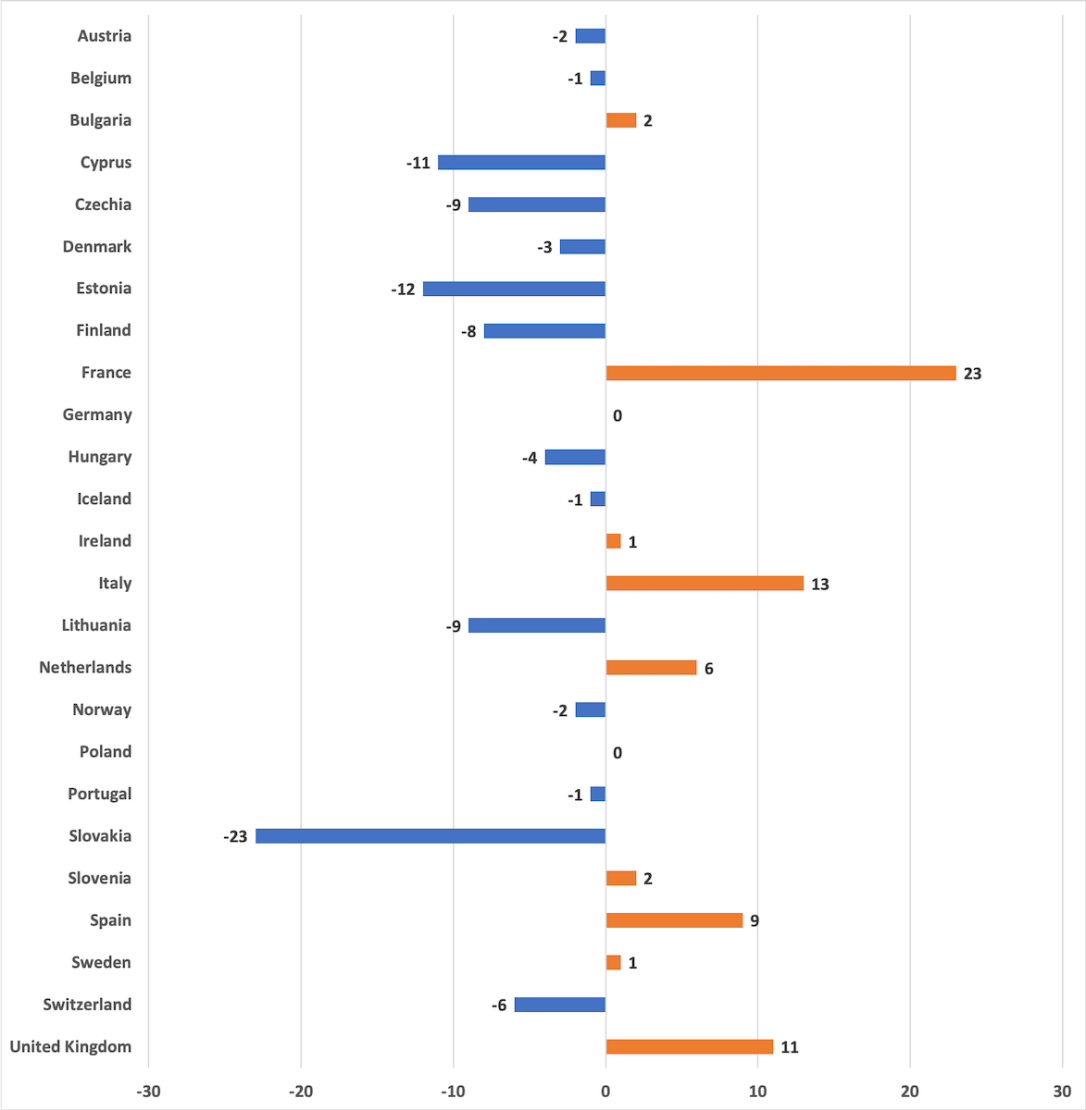
First national restrictions placed before (-) or after (+) the first COVID-19 death (days).

Descriptive statistics on the 25 European countries are shown in Table 1. The spread of the COVID-19 epidemic has been fast everywhere, but our findings reveal significant differences between countries. The most impacted countries in Europe by April 14 are Italy, Spain, and France (see Table 2). All of these countries were also significantly late to implement national restrictions. For example, Italy placed national restrictions almost 2 weeks after the first COVID-19 incident (see Figure 1). France already had 1 death case in February and was the slowest to react nationwide. It is highly likely that during these days the virus was able to spread fast in the population, which explains the later mortality figures.

**Table 1:** Descriptive statistics on 25 European countries selected for the analysis.

Country	Population density (persons per square km)	Old-age dependency ratio, %	Male, %	Household size average, n	Older adults (≥65 years) living with children, %	Life expectancy at birth (years)	Health care expenditure/ inhabitant (euros)	Tourist arrivals (millions), n	Perceived sociability	Institutional trust
Austria	107	28.2	49.2	2.2	6.7	81.8	4248.0	30.8	2.9	25.8
Belgium	375	29.5	49.3	2.9	9.6	81.7	3744.0	9.1	2.7	24.6
Bulgaria	64	33.2	48.5	2.6	16.1	75.0	556.0	9.3	2.8	10.8
Cyprus	94	23.8	48.8	2.8	14.1	82.9	1474.0	3.9	2.4	17.9
Czechia	138	30.4	49.2	2.3	4.6	79.1	1193.0	10.6	2.6	22.2
Denmark	138	30.6	49.8	2.6	2.6	81.0	5014.0	12.7	2.9	30.7
Estonia	30	31.0	47.2	2.5	14.3	78.5	1072.0	3.2	2.5	24.0
Finland	18	35.1	49.4	2.4	6.1	81.8	3727.0	3.2	2.7	30.6
France	106	32.5	48.3	2.2	4.2	82.9	3847.0	89.3	2.9	21.0
Germany	235	33.2	49.3	2.6	6.3	81.0	4271.0	38.9	2.7	26.2
Hungary	107	29.3	47.8	2.4	11.9	76.2	853.0	17.6	2.5	23.1
Iceland	4	21.3	51.2	3.0	12.1	82.9	4539.0	2.3	2.9	27.3
Ireland	71	21.6	49.5	2.6	12.3	82.3	4242.0	10.9	2.7	23.1
Italy	203	35.7	48.7	2.7	14.5	83.4	2475.0	61.6	2.9	18.1
Lithuania	45	30.4	46.4	2.4	10.5	76.0	899.0	2.8	2.6	21.1
Netherlands	504	29.5	49.7	2.4	2.3	81.9	4274.0	18.8	2.8	28.2
Norway	17	26.4	50.4	2.6	4.0	82.8	6730.0	5.7	2.9	32.3
Poland	124	26.4	48.4	3.1	27.9	77.7	731.0	19.6	2.6	17.6
Portugal	113	33.9	47.2	2.6	15.8	81.5	1632.0	16.2	2.6	18.8
Slovakia	112	23.5	48.8	2.8	20.3	77.4	1061.0	2.3	2.4	15.8
Slovenia	103	30.5	49.9	3.2	31.1	81.5	1657.0	4.4	2.7	17.6
Spain	93	29.5	48.6	3.0	28.3	83.5	2159.0	82.8	2.7	18.8
Sweden	25	31.9	50.3	2.5	2.8	75.9	5123.0	7.4	2.9	28.0
Switzerland	214	27.8	49.6	2.8	5.7	83.8	8841.0	10.4	2.8	30.0
United Kingdom	274	28.9	49.4	2.3	5.6	81.3	3566.0	36.3	2.7	24.4

**Table 2:** Descriptive statistics on coronavirus disease mortality and start of national restrictions in 25 European countries.

Country	Deaths, n	Deaths/1 million inhabitants, n	National restrictions				
			Public events	Curfew	Land borders	Restaurants	Schools
Austria	384	43	March 10	March 16	March 14	March 16	March 16
Belgium	4157	361	March 10	March 17	March 20	March 14	March 16
Bulgaria	35	5	March 13	March 21	March 20	March 13	March 3
Cyprus	12	14	March 3	March 24	March 15	March 16	March 11
Czechia	161	15	March 13	March 16	March 16	March 14	March 13
Denmark	299	51	March 11	N/A^a^	March 14	March 18	March 16
Estonia	31	23	March 3	N/A	March 17	N/A	March 13
Finland	64	12	March 13	N/A	March 19	March 30	March 18
France	15,729	235	March 9	March 23	March 17	March 15	March 16
Germany	3294	40	March 9	N/A	March 16	March 20	March 13
Hungary	122	12	March 11	March 28	March 17	March 17	March 16
Iceland	8	22	March 16	N/A	N/A	N/A	March 16
Ireland	406	83	March 12	N/A	N/A	March 22	March 13
Italy	21,067	349	March 9	March 9	March 9	March 21	March 5
Lithuania	29	10	March 13	N/A	March 16	March 16	March 12
Netherlands	2945	170	March 12	N/A	March 17	March 15	March 16
Norway	139	26	March 12	N/A	March 16	March 12	March 12
Poland	263	7	March 14	N/A	March 15	March 14	March 12
Portugal	567	55	March 20	N/A	N/A	March 22	March 16
Slovakia	2	0	March 10	N/A	N/A	N/A	March 9
Slovenia	56	27	March 16	N/A	N/A	March 16	March 16
Spain	18,056	385	N/A	N/A	N/A	March 15	March 12
Sweden	1033	101	N/A	N/A	N/A	N/A	March 17
Switzerland	1174	137	February 28	N/A	March 17	March 16	March 13
United Kingdom	12,107	182	March 16	March 23	N/A	March 20	March 20

^a^N/A: not applicable.

Our multilevel linear regression models analyzed the daily mortality in 25 countries (Tables 3 and 4). The fixed effect of time was a significant predictor of mortality in all of the models, indicating the increasing trend in deaths during the crisis period. According to the random part of our models, however, there was a between-country variation in this trend. In addition to a within-country change, we found that between-country factors significantly predicted mortality. Model 1 shows that the perceived sociability predicted higher daily mortality. Model 2 shows that late restrictions were associated with higher numbers of COVID-19 deaths. Model 3 shows that institutional trust was negatively associated with daily COVID-19 mortality figures. Of our control variables, population density, life expectancy at birth, health care expenditure per inhabitant, high tourist arrival, and the length of the follow-up period were positively associated with daily mortality, yet the significance of these associations varied between models.

**Table 3:** Multilevel mixed effects linear regression models predicting daily COVID-19 mortality in 25 European countries (fixed part).

Variables			Model 1						Model 2						Model 3				
			b		95% CI		*P* value		b		95% CI		*P* value		b		95% CI		*P* value
Constant			6.81		4.05-9.56		<.001		5.75		3.15-8.34		<.001		5.29		2.67-7.90		<.001
**Within-country effects**																			
	Time	0.16		0.11-0.22		<.001		0.16		0.10-0.22		<.001		0.16		0.10-0.22		<.001	
**Between-country effects**																			
	Perceived sociability	7.04		0.25-13.83		.04		N/A^a^		N/A		N/A		N/A		N/A		N/A	
	National restrictions after first death	N/A		N/A		N/A		2.55		1.08-4.02		.001		N/A		N/A		N/A	
	Institutional trust	N/A		N/A		N/A		N/A		N/A		N/A		–0.42		–0.65 to –0.19		<.001	
	Population	0.02		–0.03 to 0.08		.42		0.02		–0.03 to 0.07		.39		–0.02		–0.08 to 0.05		.60	
	Population density	0.00		0.00-0.01		.04		0.00		0.00-0.01		.20		0.00		0.00-0.01		.048	
	Old-age dependency ratio	–0.04		–0.29 to 0.20		.73		0.03		–0.20 to 0.26		.80		–0.04		–0.26 to 0.18		.73	
	Country household size average	0.96		–1.00 to 2.92		.34		0.98		–0.94 to 2.91		.32		–0.55		–2.45 to 1.35		.57	
	Life expectancy at birth	0.27		–0.01 to 0.54		.06		0.37		0.14-0.59		.002		0.29		0.05-0.52		.02	
	Health care expenditure per inhabitant	–0.60		–1.28 to 0.07		.08		–0.25		–0.59 to 0.08		.14		0.46		0.03-0.88		.03	
	High tourist arrival	0.65		–0.62 to 1.93		.32		1.11		–0.10 to 2.33		.07		2.12		0.40-3.83		.02	
	The length of follow-up period	0.13		0.06-0.20		.001		0.12		0.05-0.19		.001		0.19		0.12-0.26		<.001	

^a^N/A: not applicable.

**Table 4:** Multilevel mixed effects linear regression models predicting daily COVID-19 mortality in 25 European countries (random part).

Variables	Model 1		Model 2		Model 3		
	SD	95% CI	SD	95% CI		SD	95% CI
Time	0.10	0.07-0.15	0.10	0.07-0.15		0.11	0.07-0.15
Constant	3.58	2.55-5.00	3.60	2.61-4.97		3.59	2.59-4.97

**Figure 2:**
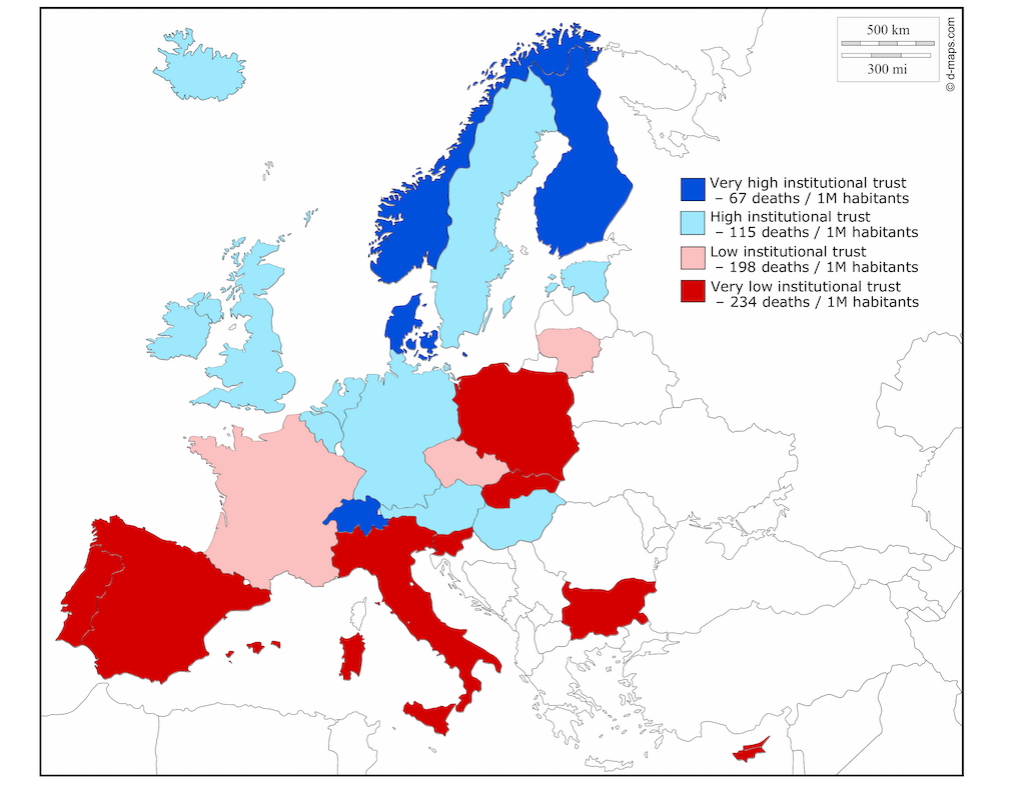
Mean deaths per million inhabitants by countries' level of institutional trust.

**Figure 3:**
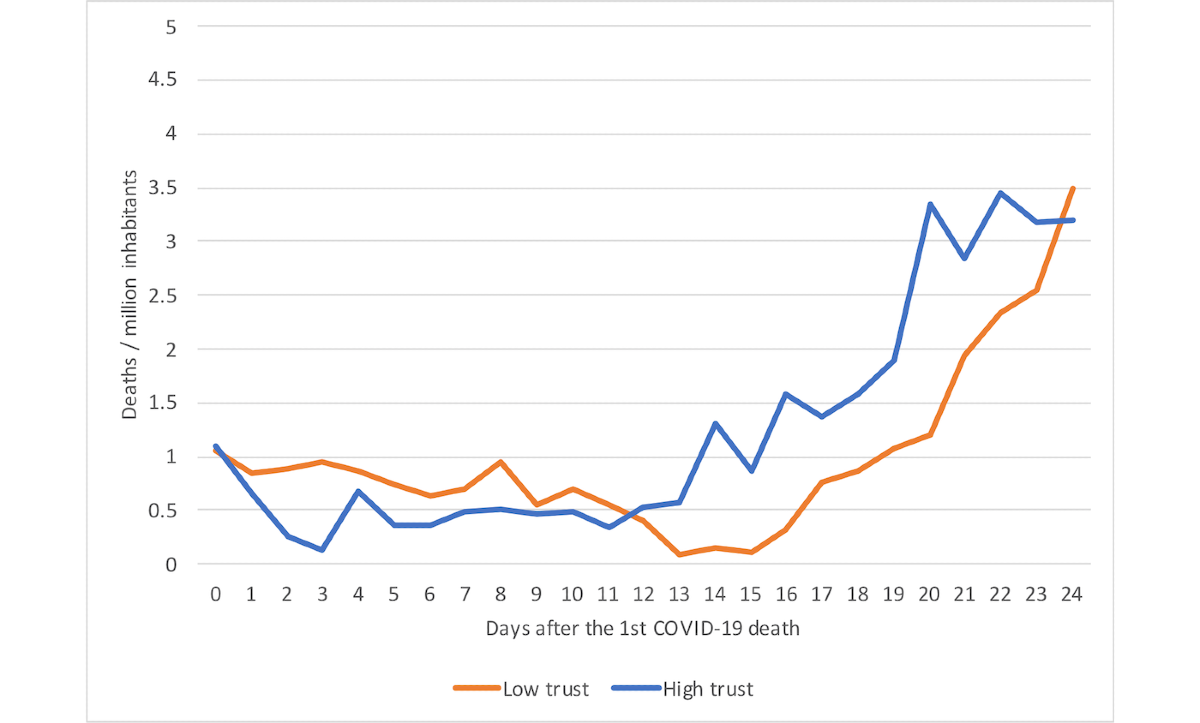
Deaths per day after first COVID-19 death in low- and high-trust countries. COVID-19: coronavirus disease.

**Figure 4:**
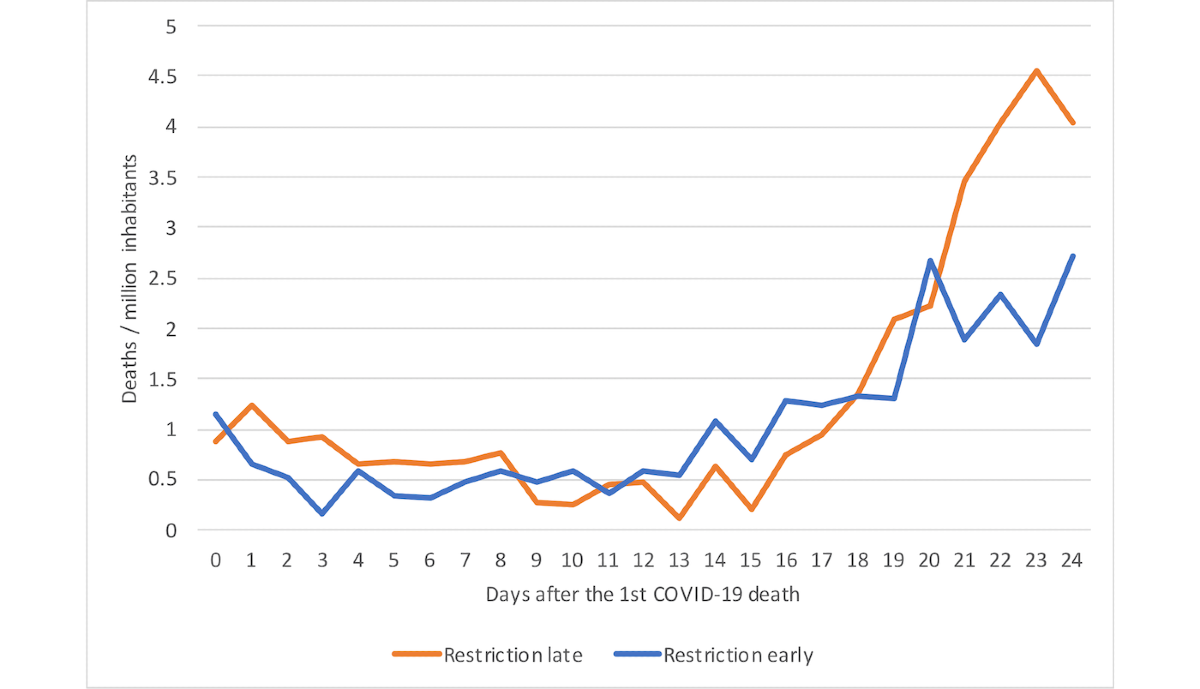
Deaths per day after first COVID-19 death in countries reacting late and early. COVID-19: coronavirus disease.

The final part of the analysis focused on the role of institutional trust and reaction time. Figure 2 shows the map of Europe and the average number of deaths per million inhabitants in the analyzed 25 countries categorized in four country groups based on their level of institutional trust. The map demonstrates that those countries with low institutional trust have more deaths per million inhabitants on average compared to countries with high trust. We analyzed the difference between countries with low vs high perceived institutional trust using a random effects regression model. Figure 3 shows development after the first COVID-19 death case in low- and high-trust countries. There are no statistically significant differences between the curves. Both curves indicate increases in mortality 2 weeks after the first COVID-19 death case, and there were no statistically significant differences between them. Figure 4 shows deaths per million inhabitants for countries reacting late and early. We can see how the number of deaths per day varied in the 24 days following the first national restrictions, and there is a statistically significant difference between the curves. Increases in mortality were more rapid in those countries reacting late than those reacting early. For example, 23 days after the first COVID-19 death there were 2.5 times more deaths in late-reacting countries (4.56 deaths/million, 95% CI 3.34-5.78) than in early reacting countries (1.83 deaths/million, 95% CI 1.02-2.65).

## Discussion

The starting points for this study were the major country differences observed in COVID-19 mortality and the related societal and cultural differences, as well as how people act in different societies during the current crisis situation. We analyzed social risk factors that may explain the spread of the COVID-19, restrictions and control measures, and institutional trust in an attempt to understand the prevailing country differences.

Our analysis showed that there were major variations in reactions to the worldwide epidemic. We were able to show that mortality was significantly associated with the studied social factors. Perceived sociability was associated with higher COVID-19 mortality even after adjusting for a number of control factors. This might be important in understanding why the virus has been able to spread so fast in some countries such as Italy, which also has a dense population. The results also reflect previous cross-cultural findings showing that Italians and Spanish people have smaller social, personal, and intimate distances compared to many other European nations [8]. These countries also have strong intergenerational ties, which may explain why so many older adults got sick [31].

One of the key points of our analysis is, however, that the COVID-19 mortality is tied to societal processes. We found major differences in how fast countries were reacting to the COVID-19 outbreak. Compared to China, European countries had time to react, yet national restrictions were placed late. Those countries that are now being hit the hardest by the disease were also the ones that were slowest to react nationwide, most notably Italy, Spain, and France. Our models showed that late national restrictions predicted a higher number of deaths. Despite the unity provided by the European Union, European countries were not working together against the emerging disease threat, and the regulations progressed slowly, taking one step at a time. There were also delays in putting the restrictions into action. Some countries have also taken different strategies to the COVID-19 epidemic. In Scandinavia, for example, Sweden has adopted less restrictions than Denmark, Finland, and Norway. Sweden also had a higher number of deaths per inhabitants at the time of this writing. This example shows that even within similar neighboring countries national precautions to COVID-19 have been different.

We were able to demonstrate in our analysis that institutional trust was a protective factor. This is in line with previous studies indicating that people with higher institutional trust are more likely to follow the advice and guidelines given by the health authorities [16,17]. In our analysis, COVID-19 mortality figures have progressed differently in low-trust countries and high-trust countries. Remarkably, some low-trust countries such as Italy, Spain, and France were not only late in placing restrictions but had to place harder measures later, such as curfews, because people were simply not following the recommendations not to socialize with each other. Despite hard measures, these countries have also had to sanction disobedient citizens. For example, the Ministry of Interior in Italy reported intensive controls, and over 100,000 people were caught by the police for breaking the curfew [32].

Epidemiologists have not necessarily given enough attention to the societal and social psychological factors explaining epidemics. Although there have been virus epidemics before, the crisis caused by COVID-19 has created a unique global situation, demonstrating how poorly the previous epidemics (eg, SARS and Middle East respiratory syndrome) have prepared countries to deal with this disease [33]. What has made the COVID-19 situation unique when compared to other epidemics, has been the rapid spread of the virus and the unusually hard restrictions placed to prevent physical contact and closeness between people. As European countries in general rely on individual freedom and democracy, it is difficult to close and shutdown societies completely. It becomes crucial to understand how different societies are capable of handling the crisis situation. This is typically reflected in the literature as societal resilience, and institutional trust is an important part of it [14]. As the crisis is not over, later developments will reveal what kind of role institutional trust eventually had on the wider picture, which also involves factors related to social contacts between people and timely restrictions placed within societies. Our analysis was limited to a relatively short follow-up period and the inability to control for all possible factors involved. We also wish to note that variations across countries exist. This involves, for example, the fact that high-trust countries have adopted different societal strategies to tackle the COVID-19 crisis. Future studies should continue using social scientific evidence in the investigations of worldwide epidemics.

## Abbreviations

COVID-19: coronavirus disease
ESS: European Social Survey
R_0_: reproductive number
SARS: severe acute respiratory syndrome
